# Optimal Service Distribution in WSN Service System Subject to Data Security Constraints

**DOI:** 10.3390/s140814180

**Published:** 2014-08-04

**Authors:** Zhao Wu, Naixue Xiong, Yannong Huang, Qiong Gu

**Affiliations:** 1 School of Mathematics and Computer Science, Hubei University of Arts and Science, Xiangyang 441053, China; E-Mails: wuzhao73@163.com (Z.W.); yannonghuang@gmail.com (Y.H.); gujone@163.com (Q.G.); 2 School of Computer Science, Colorado Technical University, Colorado Springs, CO 80907, USA

**Keywords:** wireless sensor networks, data security, services composition, reliability optimization, task partition, universal generating function, genetic algorithm

## Abstract

Services composition technology provides a flexible approach to building Wireless Sensor Network (WSN) Service Applications (WSA) in a service oriented tasking system for WSN. Maintaining the data security of WSA is one of the most important goals in sensor network research. In this paper, we consider a WSN service oriented tasking system in which the WSN Services Broker (WSB), as the resource management center, can map the service request from user into a set of atom-services (AS) and send them to some independent sensor nodes (SN) for parallel execution. The distribution of ASs among these SNs affects the data security as well as the reliability and performance of WSA because these SNs can be of different and independent specifications. By the optimal service partition into the ASs and their distribution among SNs, the WSB can provide the maximum possible service reliability and/or expected performance subject to data security constraints. This paper proposes an algorithm of optimal service partition and distribution based on the universal generating function (UGF) and the genetic algorithm (GA) approach. The experimental analysis is presented to demonstrate the feasibility of the suggested algorithm.

## 1. Introduction

Wireless Sensor Network (WSN) is validated to become an integral part of the future Internet where they extend the Internet to the physical world [1,2]. It has been imported to adapt business processes and the underlying software infrastructure quickly and flexibly to react to changes on the markets. To achieve this goal, organizations have focused on modeling, analysis and adaptation of business processes since early 2004 [3]. In recent years, WSN service system has been presented for integrating seamlessly WSN with existing, widely deployed Service-Oriented Architecture (SOA) technologies to build WSN Service Applications (WSA) [4,5]. In WSA, data stemming from WSN may influence the control flow of a business process in real-time or even trigger a business process. In these approaches, WSN is packaged as some standard Web services which can be published, located, and invoked across the Web. Therefore, these WSN services can be combined into workflows to fulfill certain tasks in a services composition way [6,7].

How to maintain the data security, reliability and performance in WSA is an important issue, which has attracted wide attention from researchers [8–10]. Great progress has been made in this field. However, little research focused on maintaining the reliability and performance at the same time ensuring the data security in WSA. Recently, there has been growing interest in this field. Methods and technologies related to data security for WSA have attracted attention because they can forecast the data security level that users will obtain from WSA [11,12]. In addition, it is helpful to analyze whether there are some data security bottlenecks in WSA.

The real and specific problem in the WSN service system is coordinated resource sharing in distributed dynamic WSN [13–15]. It is related to the SNs resource allocation issue with resource-related constraints in WSN [16,17]. The resource sharing is not primarily data exchange but rather access to SNs, software, data, and other resources, which are expressed in the form of services. This is achieved by way of collaborative problem-solving and resource-brokering strategies emerging in industry, science, and engineering.

The sharing is controlled by a WSN Services Broker (WSB) [18,19]. When the WSB receives a service request from a user, the task can be mapped into a set of atom-services (ASs) according to related business rules from domain experts. Then, these ASs can be sent into a set of sensor nodes (SNs) that are executed in parallel. After SNs finish the assigned ASs, they return the results back to the WSB and then the WSB integrates the received results into the entire task output, which is requested by the user.

The reliability and performance of WSA is of great concern. Usually, the measure of performance is the task execution time (service time) [20,21]. This index can be significantly improved using the WSB that divides a task into a set of ASs, which can be executed in parallel by some SNs. Many complicated and time-consuming tasks that could not be implemented before are currently working well under the WSN service system. The reliability can also be significantly improved using the WSB that assigns the same AS into several SNs.

The service time is a random variable affected by many factors. First, there are many SNs available in WSN service system, which have different task processing speeds. Thus, the task execution time can vary depending on which SN is assigned to execute the AS. Second, some SNs can fail when running the AS, so the execution time is also affected by the SN reliability. Similarly, the communication links in SNs can fail during the data transmission. Thus, the communication reliability influences the service time as well as data transmission speed in the communication channels.

The choice of the group of ASs assigned to the same SN can influence the total amount of data transmitted between the WSB and SN, since different ASs can use common input data. This also affects the entire service time.

The data security is also a crucial issue in WSN service system [22]. The greater number of SNs and communication channels process and transmit some data blocks the greater chances of unauthorized access (UA) to these blocks [23,24]. Different channels and SNs can have different security levels. Therefore, the distribution of ASs among the SNs affects the data security during the service execution.

Most of the previous researchers separated performance, reliability, and security into different fields and studied them individually. However, in fact, performance, reliability and security are closely related and affect each other, in particular when the WSA is implemented [25,26]. For example, when a task is divided into *n* different ASs executed by *m* SNs simultaneously, the performance is high but the reliability can be low because failure of any SN makes the entire task incomplete. This causes the task to restart, which inversely increases its execution time (*i.e.*, reduces its performance). Therefore, it is worth having some redundant SNs to execute the same AS especially for those failure-prone SNs. However, too many redundancies, even though improving the reliability, can decrease the performance by not fully parallelizing the execution of different AS and considerably reduces data security as multiple replicas of the same data are processed by different SNs, which increases chances of UA. Thus, performance, reliability and security should be studied together in the WSN service analysis.

This paper studies the optimization method that distributes service tasks into a set of SNs available in the WSN service system for maintaining the reliability, performance and data security of WSA. The paper is organized as follows. Section 2 presents the architecture of the WSN service system and the mechanisms of service partition and distribution, as well as the data security constraints. A set of assumptions are given in the end of Section 2. Section 3 presents the model of service execution time, data security, service reliability and expected performance of WSA. Section 4 presents an effective algorithm for determining the service reliability, performance and data security of WSA based on Universal Generating Function Technique (UGF). Based on this, Section 5 describes the optimization technique based on GA. In the end, the experimental analysis and conclusions are described in Sections 6 and 7, respectively.

## 2. WSN Service System

### 2.1. Architecture

**Figure 1.**
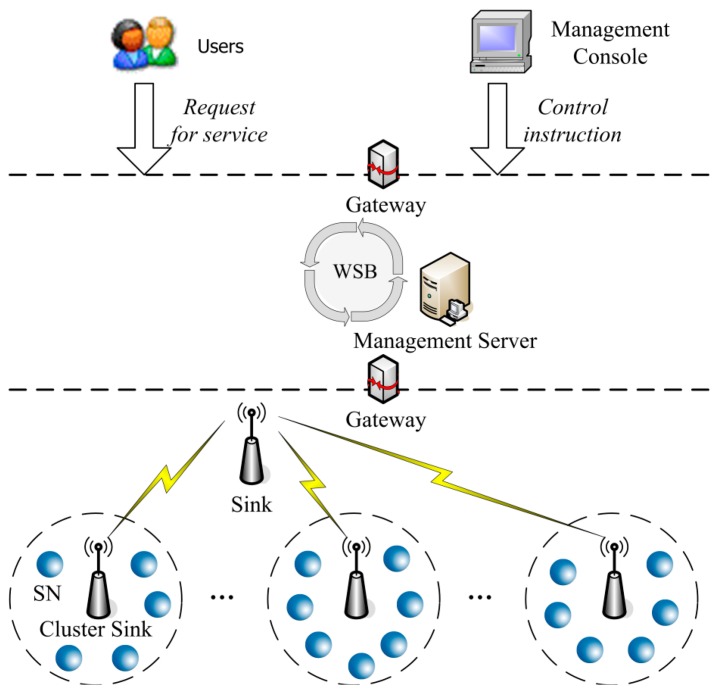
Architecture of wireless sensor network service system.

The architecture of a WSN service system can be approximated as a three-tier structure as shown in Figure 1. At the bottom of Figure 1, some redundancy SNs are deployed which compose a cluster. To assure the correctness of observed data provided by these SNs and to improve the reliability of WSA, These SNs in the same cluster are responsible for observing the same object. That is to say, at the same observation time the same observed data should be sent by these redundancy SNs for the same object.

The sink is responsible for receiving the observed data from the cluster-sinks, and sending them to the management server through the gateway. From the point of view of the data processing, the SNs can be considered as resources, because they provide the observed data of target objects to the WSN service system.

In the intermediate layer of Figure 1, the management server controls the startup, initialization, distribution and recovery of the managed sinks, cluster-sinks and nodes dynamically.

As the resource management center, the WSB is one of the important components in the management server. The WSB manages users' service requests and controls the startup, access to, and sharing of data resources located in the management server. When a user's service request is received, the WSB maps the service request into a super-service which is not a physical WSN service but a logical service at first. Then, according to some domain-specific business rules the WSB divides the super-service into a services composition which consists of a set of sub-services. Each sub-service represents a certain, specific business operations.

Unfortunately, there are usually not some physical services matching these sub-services in WSN service system. Therefore, the WSB must map each sub-service into a services composition that consists of a set of physical WSN services, *i.e.*, ASs. The next section illustrates the mapping process from a user's service request into a WSA.

### 2.2. Service Partition

Service partition is fulfilled by the WSB through a mapping process from a service request into a WSA, which is a service composition comprising a set of ASs physically. The WSB follows the related domain-specific business rules to execute the mapping process. The domain-specific business rules define the mapping relations between the service requests and the functionalities of a set of ASs, as well as their workflows. Therefore, the functionality of the generated WSA can satisfy the user's service request. By way of collaboration among ASs, the WSA can fulfill the service request from users.

**Figure 2.**
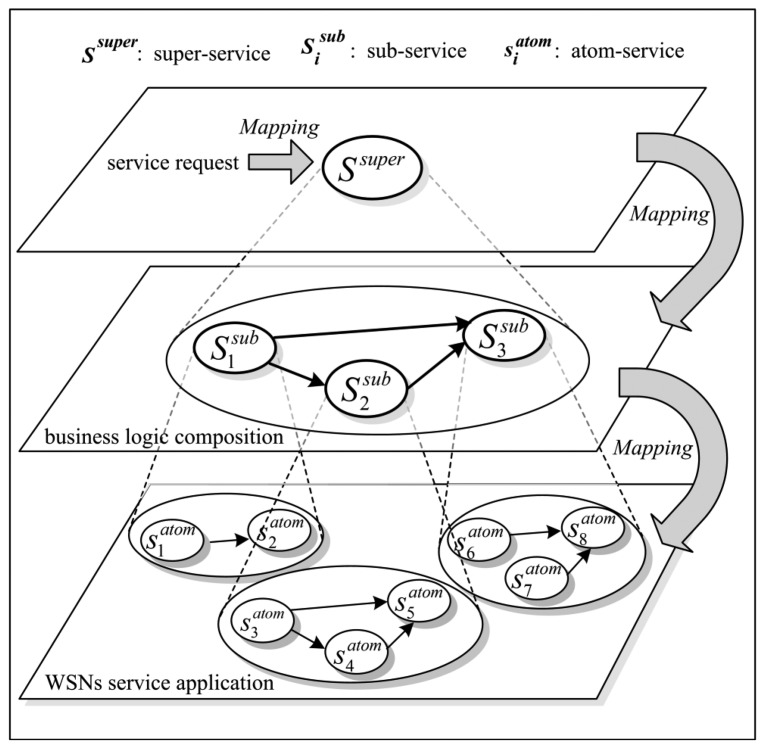
Mapping process from service request into a WSA.

Figure 2 illustrates the above mapping process which forms a three-tier structure. Each tier represents the different abstract granularities. Specifically, the top tier represents the super-service which corresponds to the service request from users; the middle tier represents the sub-services composition which corresponds to the business logic composition according to the domain-specific business rules; the bottom tier represents the ASs composition, *i.e.*, WSA, which comprises some physical WSN services.

The WSA consists of a set of ASs which should be executed by some SNs of different types. Some initiatory ASs may be located at the head of the workflow, *i.e.*, the composition structure of the WSA. Therefore, the WSB should allocate suitable SNs for them, and execute them at first. The execution of the behind ASs may require input data which should have been generated by the front ASs in the composition structure of the WSA. The execution logics of the WSA control the execution order of ASs. When a front AS was fulfilled and the result was output by a resource on which the AS was executed, the WSB transfers the received result to the next AS as it inputs data according to the execution logics, and allocates suitable resources to execute this AS. When these ASs in the WSA are all fulfilled and the final result is returned, the service request is executed completely under the control of the WSB. In the end, the final result should be transferred to the user by the WSB.

The service request is fulfilled by a WSA through the data processing at ASs and data exchange between the WSB and ASs, in fact. The data security of services is related to the successful completion of the users' service requests. The data security of sub-service is discussed in the next section.

### 2.3. Data Security of Service

The SNs and communication channels are distributed in the WSN service system. The various types of SNs with corresponding channels are of different insecurity levels. These insecurity levels are characterized by the probabilities of unauthorized access to data processed/generated by SNs. Usually, the insecurity level is determined as a product of the probability of an attack against a resource and resource vulnerability (conditional probability of the attack success given the resource is attacked). The attack probability can depend on the resource attractiveness to the attackers and can be evaluated by security experts and attack statistics. The resource vulnerability usually depends on the protection efforts made by the managers of WSN service system and WSA developers. Different models for evaluating the vulnerability of software operating systems can be found, for example, in [27,28].

In the WSN service system, the service request can be fulfilled by a set of ASs independently executed on different SNs. Each AS can be characterized by a fixed complexity, a fixed amount of output data and a fixed set of input data blocks necessary for the AS execution. Different ASs may need some common input data blocks for their execution. Some business-dependent ASs belong to the same sub-service according to the mapping process. For example, from Figure 2, one can see that the ASs $S_{1}^{\text{atom}}$ and $S_{2}^{\text{atom}}$ belong to the sub-service $S_{1}^{\text{sub}}$, and ASs $S_{3}^{\text{atom}}$, $S_{4}^{\text{atom}}$ and $S_{5}^{\text{atom}}$ belong to the sub-service $S_{2}^{\text{sub}}$, and so on. In addition, the output data block from $S_{1}^{\text{sub}}$ is the input data block of $S_{2}^{\text{sub}}$ and $S_{3}^{\text{sub}}$.

The input data block for any sub-service consists of input data blocks necessary for executing all ASs belonging to this sub-service. Execution of some ASs is associated with transferring and/or processing data. The unauthorized access to this data results in the security failure of service. The security failure occurs if sensitive data is processed by an AS or transmitted by a channel when unauthorized access to the AS or channel is accomplished.

Some data sets can be useful only in their integrity. For example, in Figure 2, only after the output data blocks from ASs $S_{3}^{\text{atom}}$ and $S_{4}^{\text{atom}}$ have been processed by $S_{5}^{\text{atom}}$ according to some certain business rules, the resulted data block output by $S_{5}^{\text{atom}}$ can be useful. In other words, each sub-service has relative-independent data security. If unauthorized access occurs only to some SNs executing a part of ASs or some channels transferring a part of data blocks from these ASs, the service security of sub-service will not become invalid completely. On the contrary, only if unauthorized access occurs to all of the SNs or all of the channels used by a sub-service, the service security of this sub-service will be invalid completely.

The SNs are involved in data exchange process. The next section presents the allocation of SNs, *i.e.*, the distribution of ASs.

## 2.4. Service Distribution

**Figure 3.**
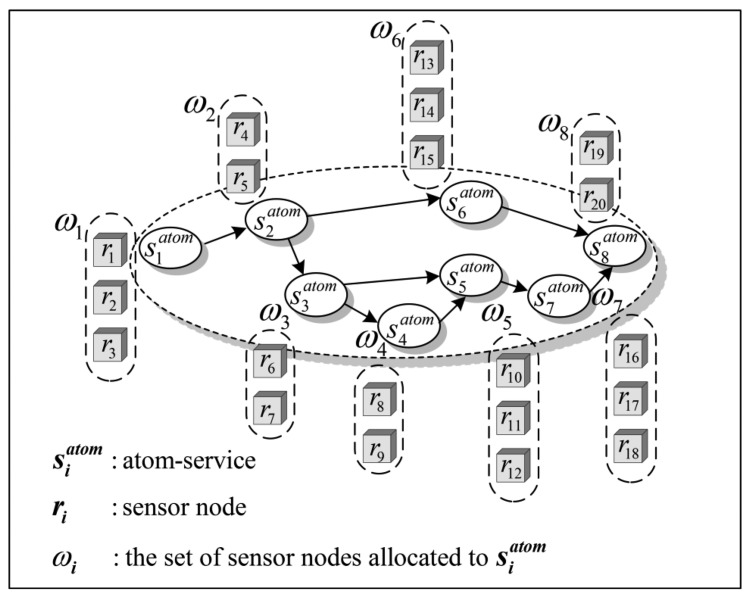
Redundant SNs allocation for ASs.

The service distribution is fulfilled by WSB through the process of SNs allocation. Usually, each sensor node can only process a single AS when it is available. On the other hand, the same AS can be assigned to several SNs in the same cluster. Considering the reliability and efficiency of WSN service system, WSB usually allocates multiple SNs for each AS to execute in parallel, which is illustrated in Figure 3.

There are 18 SNs allocated to AS $S_{1}^{\text{atom}}$ to $S_{8}^{\text{atom}}$ in Figure 3. Specifically, the set ***ω***_1_ of SNs is allocated to the AS $S_{1}^{\text{atom}}$, which can be expressed by ***ω***_1_ = {*r*_1_,*r*_2_,*r*_3_}, the set ***ω***_2_ is allocated to $S_{1}^{\text{atom}}$, ***ω***_2_ = {*r*_4_,*r*_5_}, and so on.

To improve reliability, a check mechanism, named *k* out of *n*, is introduced in WSN service system. After receiving the data blocks sent by *n* SNs in the same cluster, the cluster-sink compares them. When *k* out of *n* received data blocks are the same, where *k* ≤ *n* distinctly, the cluster-sink will regard them as the correct results and send one of them to WSB in the end. For other *k* − *n* different data blocks will be regarded as inaccurate data and be discarded by the cluster-sink, simply.

To improve efficiency, when *k* same data blocks are obtained before all of the *n* data blocks have been received, WSB makes a finished mark for this AS and cancels the execution of other SNs allocated to this AS.

The values of *k* and *n* are related to the expected service reliability from WSN service designers and the number of SNs allocated to each ASs.

The reliability and efficiency of WSN service system are improved through employing the redundant allocation of SNs and *k* out of *n* check mechanism, whereas the service cost is also improved along with the improvement of the number of redundant SNs at the same time.

Thus far, we have discussed the architecture of the WSN service system, the mechanism of service partition and distribution, as well as the data security of sub-service. For analyzing the performance, reliability and security in WSN service system, some assumptions are presented in the next section.

### 2.5. Assumptions

(1) After receiving necessary input data from WSB through the corresponding communication channel, each sensor node starts immediately processing assigned AS. Once the assigned AS is fulfilled, each sensor node sends output data to WSB through the same communication channel.
(2) Each sensor node has a given constant data processing speed and a given constant failure rate whenever it is idle, loaded or busy. In addition, the failure rates at different SNs are independent. The processing time used for an AS is proportional to its computational complexity.
(3) Each communication channel has a given constant data transmission speed and a given constant failure rate whenever it is idle, loaded or busy. In addition, the failure rates at different SNs are independent. The data transmission time is proportional to the amount of data transferred between the WSB and sensor node.
(4) All of the sub-services in a WSA and the entire ASs in a sub-service are executed serially, *i.e.*, not concurrently.
(5) The WSB is fully reliable. Comparing with the processing time of AS, the time of service processing by the WSB can be negligible.
(6) The unauthorized accesses to different SNs or communication channels are independent events. The probability of unauthorized access depends on the protection level of SNs and their communication channel and does not depend on the amount of processed data and type of services processed by ASs.

According to the above assumptions, the measurements methods on performance, reliability and security in WSN service system are presented in the following sections.

## 3. Model

### 3.1. Service Execution Time

Assume that all of the ASs within a WSA can be executed independently through service partition by the WSB. Each AS fulfills its functionality through processing the input data exported by its anterior ASs in the composition structure of a WSA. Let the computational complexity of each AS be *c_j_*, and the amount of the input data of each AS *j* be *I_j_*, while the amount of the output data of each AS *j* is *O_j_*. The completion time of each AS *j* depends on the processing speed of the SNs and the transmission speed of the communication channels of these SNs. Let the processing speed of the SN *l* be *x_l_*, and the transmission speed of its communication channels be *s_l_*. The execution time of AS is defined as the time from the beginning of input data transmission from the WSB to the SNs, on which the AS is executed, to the end of transmission of the final output data from these ASs to the WSB. Therefore, according to Assumption (1), the random time *t_jl_* of AS *j* completion by the SN *l* can take two possible values

(1) $$t_{jl}={\hat{t}}_{jl}={\hat{t}}_{jl}^{\text{processing}}+{\hat{t}}_{jl}^{\text{transmission}}$$

where ${\hat{t}}_{jl}^{\text{processing}}=\frac{c_{j}}{x_{l}}$ and ${\hat{t}}_{jl}^{\text{transmission}}=\frac{I_{j}+O_{j}}{s_{l}}$.

According to the *k* out of *n* check mechanism presented in Section 2.4, if the number of the same output data transferred from SNs to the WSB is equal to *k*, then the AS is successful and the WSB will immediately cancel the other execution of this AS by other SNs, and record the execution time of cancelled ASs as zero. Assume that the AS *j* is assigned to a set ***ω**_j_* of SNs. Therefore, the total service processing time of the AS *j* takes the following form

(2) $$t_{j,{\text{ω}}_{j}}={\hat{t}}_{j,{\text{ω}}_{j}}=\underset{l\in {\text{ω}}_{j}}{\text{max}}({\hat{t}}_{jl})$$

On the contrary, if the number of the same output data transferred from SNs to the WSB is less than *k*, then the WSB considers that this AS is failed, and records the AS execution time as ∞.

Following [29], we adopt the Crow/AMSAA reliability model in which failure intensity *h*(*τ*) for each SN is expressed as a function of development test time *τ* as follows:

(3) $$h(\tau )=\lambda \beta \tau^{\beta -1}$$

where *λ* and *β* are model parameters (*λ* > 0, 0 < *β* < 1). This model is based on the assumption that the failures during time *τ* occur as a non-homogeneous Poisson process with decreasing failure intensity. After the completion of reliability growth testing (RGT) at time *τ*, subsequent failures occur in accordance with a homogeneous Poisson process at a constant rate of *h*(*τ*). It is also assumed that failure of inter-arrival times are exponentially distributed after the completion of RGT. Therefore, the mean inter-arrival time (MTTF) can be expressed as

(4) $$\text{MTTF}=h^{-1}(\tau )=\frac{\tau^{1-\beta }}{\lambda \beta }$$

For non-repairable elements, their reliability *r* after performing RGT during time *τ* can be expressed by the following function of time *t*:

(5) $$r(t)=e^{-h(\tau )t}=e^{-\lambda \beta \tau^{\beta -1}t}$$

According to Assumptions (2) and (3), SNs and their communication channels have the constant failure rates *λ_l_* and *π_l_*, respectively. Therefore, the parameters of the above equations can be as: *h*(*τ*) = *λ_l_* + *π_l_*, and *t* = $t={\hat{t}}_{jl}$ in this paper.

AS *j* can be successfully completed by SN *l*, if this SN and its communication link do not fail before the end of AS execution. For constant failure rates of SN *l* and its communication link (Assumptions (2) and (3) presume exponential distribution of time to failure), one can obtain the conditional probability of AS *j* success given both SN *l* & link *l*, *i.e.*, the reliability of AS *j*, are available at the beginning of the AS execution as

(6) $$r({\hat{t}}_{jl})=p_{jl}({\hat{t}}_{jl})=e^{-(\lambda_{l}+\pi_{l}){\hat{t}}_{jl}}$$

These give the conditional distribution of the random AS *j* execution time *t_jl_* as: Pr(*t_jl_* = *t̂_jl_*) = *p_l_*(*t̂_jl_*) and Pr(*t_jl_* = ∞) = 1 − *p_l_*(*t̂_jl_*).

According to Assumption (4) that all of the sub-services in a WSA and the entire ASs in a sub-service are executed serially, for any one of sub-service *i* comprising *m* ASs, *i.e.*, the set of ASs ***σ**_i_* (|***σ**_i_*|= *m*), its random completion time takes the form

(7) $$t_{i,{\text{σ}}_{i}}={\hat{t}}_{i,{\text{σ}}_{i}}=\sum_{j=1}^{m}{\hat{t}}_{j,{\text{ω}}_{j}}$$

Similarly, for the WSA comprising *h* sub-services, *i.e.*, the set of sub-services ***φ*** (|***φ***|= *h*), its total completion time takes the form

(8) $$T_{\text{φ}}={\hat{T}}_{\text{φ}}=\sum_{i=1}^{h}{\hat{t}}_{i}=\sum_{i=1}^{h}\sum_{j=1}^{m}{\hat{t}}_{j,{\text{ω}}_{j}}$$

Having the distributions of each random time *t̂_j,_****_ω_****__j__* one can obtain the distribution of the entire WSA execution time *T* (probability mass function of discrete variable *T*) in the form of pairs (*T_f_*, *P_f_*), 0≤ *f* ≤ *F*, where *T_f_* is *f*-th realization of *T*, *P_f_* = Pr(*T* = *T_f_*), and *F* is total number of different realizations of *T*.

### 3.2. Data Security in WSN Service System

When AS *j* is executed by a set of SNs ***ω**_j_*, the unauthorized access to any one of the SNs, as well as its communication channel, belonging to the set ***ω**_j_* results in the security failure of this AS. Let the security of the SN *l* and its communication channel is *y_l_*, the probability of security failure of the AS *j* assigned to the set of SNs ***ω**_j_* can be represented as

(9) $$f_{j,{\text{ω}}_{j}}=1-\prod_{l}{\left(y_{l}\right)}^{1(l\in {\text{ω}}_{j})}$$

If all of the ASs in a sub-service have been compromised by unauthorized access, the unauthorized access to this sub-service succeeds. Assume that a sub-service *i* comprises a set of ASs ***σ**_i_* The probability of security failure of the sub-service *i* can be obtained as

(10) $$f_{i,{\text{σ}}_{i}}=\prod_{j}{\left(f_{j}\right)}^{1(j\in {\text{σ}}_{i})}=\prod_{j}{\left(1-\prod_{l}{\left(y_{l}\right)}^{1(l\in {\text{ω}}_{j})}\right)}^{1(j\in {\text{σ}}_{i})}$$

Similarly, for a WSA comprising a set of sub-services ***φ***, its probability of security failure can be obtained as

(11) $$f_{\text{φ}}=\prod_{i}{\left(f_{i}\right)}^{1(i\in \text{φ})}=\prod_{i}{\left(\prod_{j}{\left(f_{j}\right)}^{1(j\in {\text{σ}}_{i})}\right)}^{1(i\in \text{φ})}=\prod_{i}{\left(\prod_{j}{\left(1-\prod_{l}{\left(y_{l}\right)}^{1(l\in {\text{ω}}_{j})}\right)}^{1(j\in {\text{σ}}_{i})}\right)}^{1(i\in \text{φ})}$$

Obviously, the risk of security failure of a WSA is increased along with the increase of the number of SNs which are unauthorized accessed. The security requirement of WSN service system can be defined as a threshold $f_{\text{φ}}^{*}$ of security failure probability. It can be obtained from the security experts or the designers of the WSN service system. When the security failure probability *f*_***φ***_ is less than $f_{\text{φ}}^{*}$, the WSN service system is considered to be secure.

### 3.3. Service Reliability and Expected Performance

In order to evaluate the service reliability and performance of the WSN service system, the service execution time has been defined in Section 3.1. According to the performance concept in [30,31], the system reliability *R*(T*) is defined as a probability that the correct output is produced in time less than T*. This index can be obtained as

(12) $$R(T^{*})=\sum_{f=1}^{F}P_{f}1(T_{f}<T^{*})$$

The number of fulfilled service requests over a fixed time, *i.e.*, the average service performance, is an important index in the WSN service system. The service reliability is defined as the probability that it produces correct outputs without respect to the service time, which can be referred as *R*(∞). Given that the service does not fail, the conditional expected service time *T̃* can be considered as the measurement of service performance. The index can be obtained as

(13) $$\tilde{T}=\sum_{f=1}^{F}T_{f}P_{f}/R(\infty )$$

Summarily, in our architecture of WSN service system, the service task, *i.e.*, super-service, corresponding to a given users' service request is partitioned into *h* sub-services (represented by the set ***φ***, |***φ***|= *h*) according to some domain-specific rules. Then, each sub-service *i* is partitioned into *m* ASs (represented by the set ***σ**_i_*, 1 ≤ *i* ≤ *h*) according to some domain-specific rules. On this basis, each AS *j* is distributed among a set of SNs (represented by the set ***ω**_j_*, 1 ≤ *j* ≤ *m*, | ***ω**_j_* | = *n*). Each SN has the constant processing speed *x_l_*, the independent security *y_l_* and the constant failure rates ***λ**_l_*. The above partitions and distribution determine the service reliability, performance and security of a WSA. Therefore, the optimal service distribution, *i.e.*, the distribution of all of the ASs among the SNs in WSN service system subject to data security constraints, can come down to two optimization problems:

(1) How to find the distribution ***ω**_j_* of each AS *j* 1 ≤ *j* ≤ *m* in the set of sub-services ***σ**_i_* 1 ≤ *i* ≤ *h* maximizing *R*(T*) for a given T* subject to the data security constraint $f_{\text{φ}}\leq f_{\text{φ}}^{*}$. It can be formalized as:
  $$\{\begin{array}{lll} & \max R(T*) & \\ \text{s}.\text{t}. & f_{\text{φ}}\leq f_{\text{φ}}^{*} & {\text{ω}}_{j}(1\leq j\leq m),{\text{σ}}_{i}(1\leq i\leq h) \end{array}$$
(2) How to find the distribution ***ω**_j_* of each AS *j* 1 ≤ *j* ≤ *m* in the set of sub-services ***σ**_i_* 1 ≤ *i* ≤ *h* minimizing *T̃* subject to the service reliability constraint *R*(∞) ≥ R* and the data security constraint $f_{\text{φ}}\leq f_{\text{φ}}^{*}$. It can be formalized as:
  $$\{\begin{array}{lll} & \min\tilde{T} & \\ \text{s}.\text{t}. & R(\infty )\geq R* & {\text{ω}}_{j}(1\leq j\leq m),\;{\text{σ}}_{i}(1\leq i\leq h)\\ & f_{\text{φ}}\leq f_{\text{φ}}^{*} & {\text{ω}}_{j}(1\leq j\leq m),{\text{σ}}_{i}(1\leq i\leq h) \end{array}$$

In solving the above two optimization problems, determining the Probability Mass Function (PMF) of the service time is primary. The following section proposes an algorithm based on the universal generating function (u-function) technique for determining the PMF of the service time for arbitrary service partition and distribution.

## 4. Algorithm for Determining the PMF of Service Time

Since the service time can take different values, the WSA should be considered as a multi-state system (MSS) [32] with performance depending on combination of states of its elements. In other words, the WSA can have different performance levels corresponding to different combinations of available and failed SNs with different processing speeds and failure rates, as well as their communication channels with different data transmission speeds and failure rates.

Generally, the methods of MSS reliability and performance assessment can be divided into four different types [33]: (1) an extension of the Boolean models to the multi-valued case; (2) the stochastic process (mainly Markov and semi-Markov) approach; (3) the Monte-Carlo simulation technique; (4) the u-function approach [34]. The first three approaches have some disadvantages, either the applicability of only small-scale MSS or too much time consumed for executing model. On the contrary, the u-function technique is fast enough, and can be applied in the assessment of reliability and performance of a large-scale MSS. This is because that u-function technique allows one to find the performance distribution of the entire MSS based on the performance distributions of its elements by using a fast algebraic procedure.

For the above reasons, we choose u-function technique to determine the PMF of the service time for arbitrary service partition and distribution. The next section presents the expression of the PMF of service time base on the u-function technique.

### 4.1. Expression of the PMF of Service Time

The procedure used in this paper for the evaluation of the service time distribution is based on the UGF technique, which was introduced in [35], and which proved to be very effective for the reliability evaluation of different types of multi-state systems [36].

The service time of a WSA is a random discrete variable *T*. Assume that *T* has *K* possible values, *i.e.*, *K* possible states *T*_1_,*T*_2_,…,*T*_k_. Let *p_k_* is the probability when *T* is in the state *T_k_*, 1 ≤ *k* ≤ *K*. According to the UGF technique, the probability distribution of the service time of a WSA can be obtained using a formal operator *z* that resembles the procedure of the product of polynomials. The u-function representing the PMF of the random discrete service time *T* can be defined as a polynomial

(14) $$u(z)=\sum_{k=1}^{K}p_{k}z^{T_{k}}$$

Each term coefficient of the polynomial in Equation (14) denotes the probability that the performance of WSA takes on determinate state *k*, while the corresponding exponent denotes the performance corresponding to state *k*. The representation (14) is named the generating function (or z-transform). In general, z-transform of any random discrete service time that has the PMF *p_k_*, *T_k_* takes the form (14). The essence of z-transform representation lies in relating the possible values of the variable with the probabilities that the random variable takes these values.

Based on the above definition, the u-function representing the PMF of the completion time for an AS *j* assigned to SN *l* can take the form of

(15) uj,{l}(z)=p(t^jl)lzt^jl+[1−p(t^jl)l]z∞

According to Equation (2), the total completion time of the AS *j* assigned to a pair of SNs *l*_1_ and *l*_2_ is equal to the maximum of completion times for different SNs. In order to obtain the u-function representing the PMF of this time, a composition operator ⊗_max_ with ⊗_max_(*t̂_jl_*__1,__*t̂_jl_*__2__) = max(*t̂_jl_*__1,__*t̂_jl_*__2__) should be used

(16) $$\begin{array}{c} u_{j,\{l_{1},l_{2}\}}(z)=u_{j,\{l_{1}\}}(z)\underset{\max}{\text{⊗}}u_{j,\{l_{2}\}}(z)=p_{l_{1}}({\hat{t}}_{jl_{1}})p_{l_{2}}({\hat{t}}_{jl_{2}})z^{\max({\hat{t}}_{jl_{1},}{\hat{t}}_{jl_{2}})}+[1-p_{l_{1}}({\hat{t}}_{jl_{1}})]p_{l_{2}}({\hat{t}}_{jl_{2}})z^{{\hat{t}}_{jl_{2}}}\\ +[1-p_{l_{2}}({\hat{t}}_{jl_{2}})]p_{l_{1}}({\hat{t}}_{jl_{1}})z^{{\hat{t}}_{jl_{1}}}+[1-p_{l_{1}}({\hat{t}}_{jl_{1}})][1-p_{l_{2}}({\hat{t}}_{jl_{2}})]z^{\infty } \end{array}$$

The u-function technique provides a recursive computation approach to efficiently obtain the PMF. For a AS *j*, 1 ≤ *j* ≤ *m*, assigned to a set of SNs ***ω**_j_* = {*l*_1_,*l*_2_, ⋯, *l_n_*}, the u-function representing the PMF of the total completion time can be obtained recursively

(17) $$\begin{array}{l} u_{j,\{l_{1},l_{2}\}}\left(z\right)=u_{j,\{l_{1}\}}\left(z\right)\underset{\max}{\text{⊗}}u_{j,\{l_{2}\}}\left(z\right),\\ u_{j,\{l_{1},l_{2},l_{3}\}}\left(z\right)=u_{j,\{l_{1},l_{2}\}}\left(z\right)\underset{\max}{\text{⊗}}u_{j,\{l_{3}\}}\left(z\right),\\ \cdots ,\\ u_{j,{\text{ω}}_{j}}\left(z\right)=u_{j,\{l_{1},l_{2},\cdots ,l_{n}\}}\left(z\right)=u_{j,\{l_{1},l_{2},\cdots ,l_{n-1}\}}\left(z\right)\underset{\max}{\text{⊗}}u_{j,\{l_{n}\}}\left(z\right). \end{array}$$

Having the u-functions *u_j_*_,_*_**ω**_j__* (*z*) of each ASs *j*, according to Equation (7), we can recursively obtain the u-function representing the PMF of completion time of each sub-service *i* comprising the set of ASs ***σ**_i_* = {*j*_1_,*j*_2_, ⋯,*j_m_*} using the composition operator ⊗*_sum_* with ⊗_sum_(*t̂_j_*__1_,_*_**ω**_j__*___1___,*t̂_j_*__2_,_*_**ω**_j__*___2___) = *t̂_j_*__1_,_*_**ω**_j__*___1___ + *t̂_j_*__2_,_*_**ω**_j__*___2___

(18) $$\begin{array}{l} u_{i,\{j_{1}\}}\left(z\right)=u_{j_{1},{\text{ω}}_{j_{1}}}\left(z\right),\\ u_{i,\{j_{1},j_{2}\}}\left(z\right)=u_{i,\{j_{1}\}}\left(z\right)\underset{\mathrm{sum}}{\text{⊗}}u_{i,\{j_{2}\}}\left(z\right),\\ u_{i,\{j_{1},j_{2},j_{3}\}}\left(z\right)=u_{i,\{j_{1},j_{2}\}}\left(z\right)\underset{\mathrm{sum}}{\text{⊗}}u_{i,\{j_{3}\}}\left(z\right),\\ \cdots ,\\ u_{i,{\text{σ}}_{i}}\left(z\right)=u_{i,\{j_{1},j_{2},\cdots ,j_{m}\}}\left(z\right)=u_{i,\{j_{1},j_{2},\cdots ,j_{m-1}\}}\left(z\right)\underset{\mathrm{sum}}{\text{⊗}}u_{i,\{j_{m}\}}\left(z\right). \end{array}$$

Similarly, based on the u-functions *u_i_*_,_*_**σ**_i__* (*z*) of each sub-services, according to Equation (8), the u-function *U_**φ**_*(*z*) representing the PMF of the total completion time of a WSA comprising *h* sub-services *i* (1 ≤ *i* ≤ *h*) can be obtained recursively using the composition operator ⊗*_sum_*

(19) $$\begin{array}{l} U_{1}(z)=u_{1,{\text{σ}}_{1}}\left(z\right),\\ U_{2}(z)=U_{1}(z)\underset{\mathrm{sum}}{\text{⊗}}u_{2,{\text{σ}}_{2}}\left(z\right),\\ \cdots ,\\ U_{\text{φ}}(z)=U_{h}(z)=U_{h-1}(z)\underset{\mathrm{sum}}{\text{⊗}}u_{h,{\text{σ}}_{h}}\left(z\right). \end{array}$$

The final u-function *U_**φ**_*(*z*) represents the PMF of random completion time *T* of the service request from users in the form

(20) $$U_{\text{φ}}(z)=\sum_{f=1}^{F}P_{f}z^{T_{f}}$$

Using Equations (12) and (13), the service reliability *R*(T*) and the expected service time *T̃* can be obtained. The next section presented an algorithm for determining the service reliability, performance and security of a WSA with arbitrary service partition (***φ***,|***φ***|= *h*, ***σ**_i_*, 1 ≤ *i* ≤ *h*, |***σ**_i_*|= *m*) and distribution ***ω**_j_*, 1 ≤ *j* ≤ *m*, |***ω**_j_*|= *n*.

### 4.2. Algorithm for Determining the Service Reliability, Performance and Data Security

Given a WSA comprises the set of sub-services ***φ***, |***φ***|= *h*. Each sub-service *i* in this WSA, comprises the set of ASs ***σ**_i_*, 1 ≤ *i* ≤ *h*, |***σ**_i_*|= *m*. Each AS *j* has the amount of the input data *I_j_* and the amount of the output data *O_j_*. Each AS *j* is assigned to the set of SNs ***ω**_j_*, 1 ≤ *j* ≤ *m*, |***ω**_j_*|= *n*. Each SN *l* has the constant processing speed *x_l_*, the independent security *y_l_* and the constant failure rates ***λ**_l_*. Additionally, the communication channel of the SN *l* has the constant transmission speed *s_l_*, the independent security *y_l_* and the constant failure rates ***π****_l_.*

The algorithm for determining the service reliability, performance and security of a WSA is described as follows:

Step 1 For the given distribution ***ω**_j_*, (1 ≤ *j* ≤ *m*, |***ω**_j_*|= *n*) of each AS *j* among the SNs, determine the service processing time *t̂_jl_* and the probability *p_jl_*(*t̂_jl_*) of successful completion of AS *j* assigned to each SN *l* using Equations (1) and (6), respectively. On this basis, obtain the u-function *u_j_*_,{_*_l_*_}_(*z*) representing the PMF of completion time for the AS *j* assigned to each SN *l* using Equation (15).
Step 2 For each AS *j* assigned to the set of SNs ***ω**_j_*, determine the u-function *u_j_*_,_*_**ω**_j__* (*z*) representing the PMF of total completion time for the AS *j* assigned to the set of SNs ***ω**_j_* according to the recursive process described by Equation (17). Then, determine the probability of security failure *f_j_*_,_*_**ω**_j__* of the AS *j* using Equation (9).
Step 3 For the given service partition ***σ**_i_*, (1 ≤ *i* ≤ *h*, |***σ**_i_*|= *m*) of each sub-service *i*, determine the u-function *u_i_*_,_*_**σ**_i__* (*z*) representing the PMF of completion time of each sub-service *i* according to the recursive process described by Equation (18). Then, determine the probability of security failure *f_j_*_,_*_**σ**_i__* the sub-service *i* using Equation (10).
Step 4 For the given service partition ***φ*** (|***φ***|= *h*) of the WSA, determine the u-function *U_**φ**_*(*z*) representing the PMF of the total completion time of the WSA according to the recursive process described by Equation (19). Then, determine the probability of security failure *f_**φ**_* of the WSA using Equation (11).
Step 5 Determine the reliability *R*(T*) and the conditional expected service time *T̃* using Equations (12) and (13), respectively.

From the above algorithms described, especially (8), one can see that the time complexity of the suggested algorithm in finding the PMF is *O*(|*σ*|×|***φ***|). In other words, the time consumption of the suggested algorithm in finding the PMF is determined by the number of sub-services in a WSA and the number of ASs within each sub-service.

Using the above algorithm, the final reliability and the conditional expected service time of a WSA can be obtained, as well as the probability of security failure. On this basis, the different distribution solutions for the entire ASs in a WSA can be compared under an optimization algorithm framework. The next section proposes an optimization technique for ASs distribution subject to data security constraint.

## 5. Optimal Service Distribution Technique

Considering reasonable time limitations, an exhaustive examination of all possible solutions for the distribution of ASs among SNs is not realistic. Therefore, the optimal service distribution problem is a complicated NP complete problem. Because the quality of solution for a given AS distribution is the only information available during the search for the optimal solution, a heuristic search algorithm is used for estimating the solution quality.

The genetic algorithm (GA) has been proven to be an effective optimization tool for a large number of complicated problems in reliability engineering [37,38]. Therefore, we choose GA to solve the optimization problem of service distribution. The next section introduces the solution representation in optimal search algorithms.

### 5.1. Encoding and Decoding of Solution

In the framework of the above WSN service system, there are *h* sub-services and *m* ASs belonging to each sub-service. Each AS is assigned to *n* SNs. So, the number of the entire ASs distributed among these SNs is *h*×*m.* Furthermore, the number of SNs allocated to the entire ASs is *h*×*m*×*n.* Therefore, the service distribution problem can be considered as a problem of partitioning a set Ω of *h*×*m*×*n* SNs into a collection of *h*×*m* mutually disjoint subsets ***ω**_j_*, *i.e.*, such that

(21) $$\begin{array}{ccc} \overset{h}{\underset{i}{\text{∪}}}\overset{m}{\underset{j}{\text{∪}}}{\text{ω}}_{j}^{i}=\Omega , & {\text{ω}}_{j}^{i}\neq {\text{ω}}_{k}^{i}, & i\neq k \end{array}$$

where the set ${\text{ω}}_{j}^{i}$ represents the set ***ω**_j_* of ASs with in sub-service *i.*

In order to express concisely, let *R* = *h*×*m*×*n* and *S* = *h*×*m*, the above partition problem can be expressed as partitioning a set Ω of *R* SNs into a collection of *S* mutually disjoint subsets ***ω****_s_*, *i.e.*, such that

(22) $$\begin{array}{ccc} \overset{S}{\underset{s}{\text{∪}}}{\text{ω}}_{s}=\Omega , & {\text{ω}}_{s_{1}}\neq {\text{ω}}_{s_{2}}, & s_{1}\neq s_{2} \end{array}$$

where *S* ≤ *R* obviously.

In order to encode a feasible solution for the distribution of *S* ASs among *R* SNs, let *R* different integer numbers (from 1 to *R*) represent these SNs, and *S* different integer numbers (from *R*+1 to *R*+*S*) represent these ASs. In other words, the integer number *r* (1≤*r*≤*R*) represents any one of *R* SNs whereas the integer number *S* (*R*+1≤*s*≤*R*+*S*) represents any one of *S* ASs. Therefore, a feasible solution can be represented by a string with any permutation of *R*+*S* different integer numbers (from 1 to *R*+*S*).

The rule of permutation decoding is as follows: a set of SNs represented by any sequence of adjacent SN numbers, *i.e.*, the numbers of greater than or equal to 1 and less than or equal to *R*, execute a set of ASs represented by any sequence of adjacent AS numbers, *i.e.*, the numbers of greater than or equal to *R*+1 and less than or equal to *R*+*S*, located on the right side of this SN number sequence. In other words, all the SNs taking adjacent positions execute the same set of ASs taking adjacent positions which is located on its right side. In order to ensure that all ASs can be assigned to at least one SN, the smallest number, *i.e.*, 1, representing a SN always takes the leftmost position in the permutation. In addition, the condition of a sequence of adjacent SN numbers located on the rightmost position in the permutation implies these SNs represented by this number sequence are not assigned to any AS. In other words, they can remain idle.

Since the place of the first number, *i.e.*, the first SN, is fixed in a solution sequence, the solution depends on permutation of *R*+*S*−1 numbers. Therefore, the solution decoding procedure determines *S* mutually disjoint subsets ***ω****_s_* from any permutation of *R*+*S*−1 different numbers. On this basis, an optimal solution can be found in a random searching framework. The next section presents the crossover and mutation operations used in our random searching framework.

### 5.2. Crossover and Mutation Operations

New feasible solutions, *i.e.*, chromosomes in GA, are obtained through crossover operations and mutation operations on the solution strings. An efficient crossover procedure presented in [39] is used in this work. This procedure copies all the string elements from the first parent to the same positions of the offspring at first. Then, a fragment of this offspring is identified as a set of adjacent positions between two randomly defined positions in the string. On this basis, all the offspring numbers belonging to this fragment are reallocated in the order they appear in the second parent. In other words, for the offspring, the numbers in the defined fragment are reordered according to their order appearing in the second parent, whereas the order of the rest of offspring numbers is same as the first parent. The following is an example of the crossover procedure in which the fragment is marked in bold.


First parent:12**3****4****5****6****7****8**910Second parent:18**9****10****4****3****7****6**25First parent:12**8****4****3****7****6****5**910

The mutation operations used in our work just swaps the numbers located in two randomly chosen positions of the string.

The above crossover and mutation operations preserve solution feasibility. The optimal solution can be found through comparing the fitness of solutions generated by crossover and mutation operations. The next section proposes the searching process for the optimal service distribution based on GA.

### 5.3. Searching Process for the Optimal Solution

According to the algorithm presented in Section 4.2, the system reliability, expected service time and security corresponding to any feasible solution can be calculated. For the optimization problem (1) described in Section 3.3, the solution fitness *F* is determined as

(23) $$F=R(T*)-\text{η}(f_{\text{φ}}-f_{\text{φ}}^{*})1(f_{\text{φ}}>f_{\text{φ}}^{*})$$

For the optimization problem (2) described in Section 3.3, the solution fitness *F* is determined as

(24) $$F=\text{α}-T-\text{ɛ}\left(R*-R(\infty ))1(R(\infty )<R*\right)-\text{η}\left(f_{\text{φ}}-f_{\text{φ}}^{*}\right)1\left(f_{\text{φ}}>f_{\text{φ}}^{*}\right)$$

where ***α*** is a constant, and ***ε*** and ***η*** are penalty coefficients.

**Figure 4.**
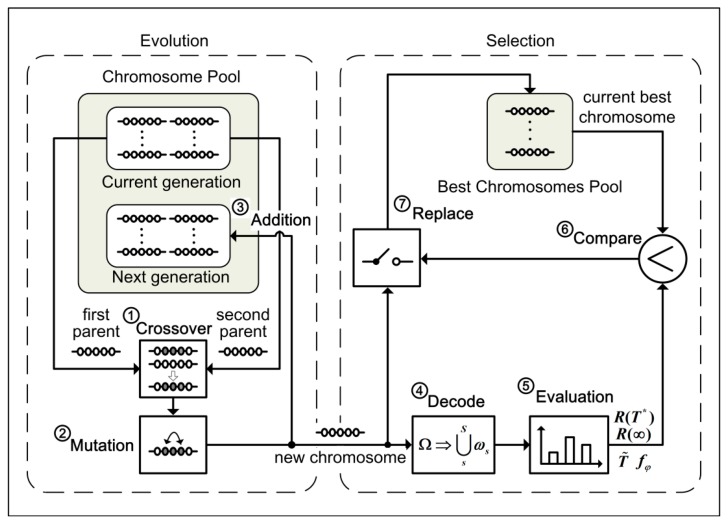
Searching process for the optimal solution.

The searching process for the optimal service distribution, illustrated in Figure 4, comprise the evolution procedure with the selection procedure on the whole. From Figure 4, one can see that the evolution procedure consists of the crossover operation, the mutation operation and addition operation corresponding to Steps 1–3 respectively, whereas the selection procedure consists of the decoding operation, the evaluating operation, the comparing operation and the replacing operation corresponding to Steps 4–7.

Before the searching process begins to work, the current generation population in the chromosome pool is initialized with *k* generating randomly chromosomes. Obviously, *k* is the size of the population. The next generation population with the same size will be generated by *k* executions of the searching process. Furthermore, some of the best chromosomes found in all searching processes will be stored in the best chromosomes' pool. Through the population evolution of some generations within the specified time, the best chromosome in the best chromosomes pool represents the optimal solution that can be found. All the steps in the searching process are described as follows.

(1) Randomly choose two chromosomes from the current population in the chromosome pool as the first parent and the second parent. Then, according to the crossover procedure described in Section 5.2, a crossover operation is performed on these two chromosomes with a specified crossover probability. As a result, a new chromosome different to its parents is generated.
(2) A mutation operation described in Section 5.2 is performed sequentially on this new generated chromosome with a specified mutation probability. Thus, a new chromosome is generated finally.
(3) Add the new generated chromosome into the next generation population in the chromosome pool.
(4) Decode the new generated chromosome to obtain the distribution ***ω****_s_* (1≤*s*≤*S*) of all ASs on SNs according to the decoding procedure in Section 5.1.
(5) Calculate the system reliability, the conditional expected service time and the probability of security failure of a WSA with ASs distribution ***ω****_s_* (1≤*s*≤*S*) on SNs using the algorithm presented in Section 4.2.
(6) According to the solution fitness defined in (23) or (24), calculate the fitness of the new generated chromosome. Then, compare it with the current worst chromosome in the best chromosome pool.
(7) If the new generated chromosome is superior to the worst one in the best chromosome pool, replace the worst one with it.

In order to examine the feasibility of our algorithm, some examples have been performed which are presented in the next section.

## 6. Experimental Analysis

Consider a WSA that uses 10 SNs distributed in a WSN service system. The processing speeds *x_l_*, the failure rates ***λ**_l_* and security probabilities *y_l_* of these SNs, and the transmission speeds *s_l_*, the failure rates ***π****_l_* of their communication channels are presented in Table 1.

The entire WSA can be mapped into three sub-services comprising six atom-services. We supposed for simplicity that all of these sub-services and atom-services are executed in series. The service partition and the computational complexities, the amount of the input data and the amount of the output data for each atom-service are presented in Table 2.

### 6.1. Experimental Environment

**Figure 5.**
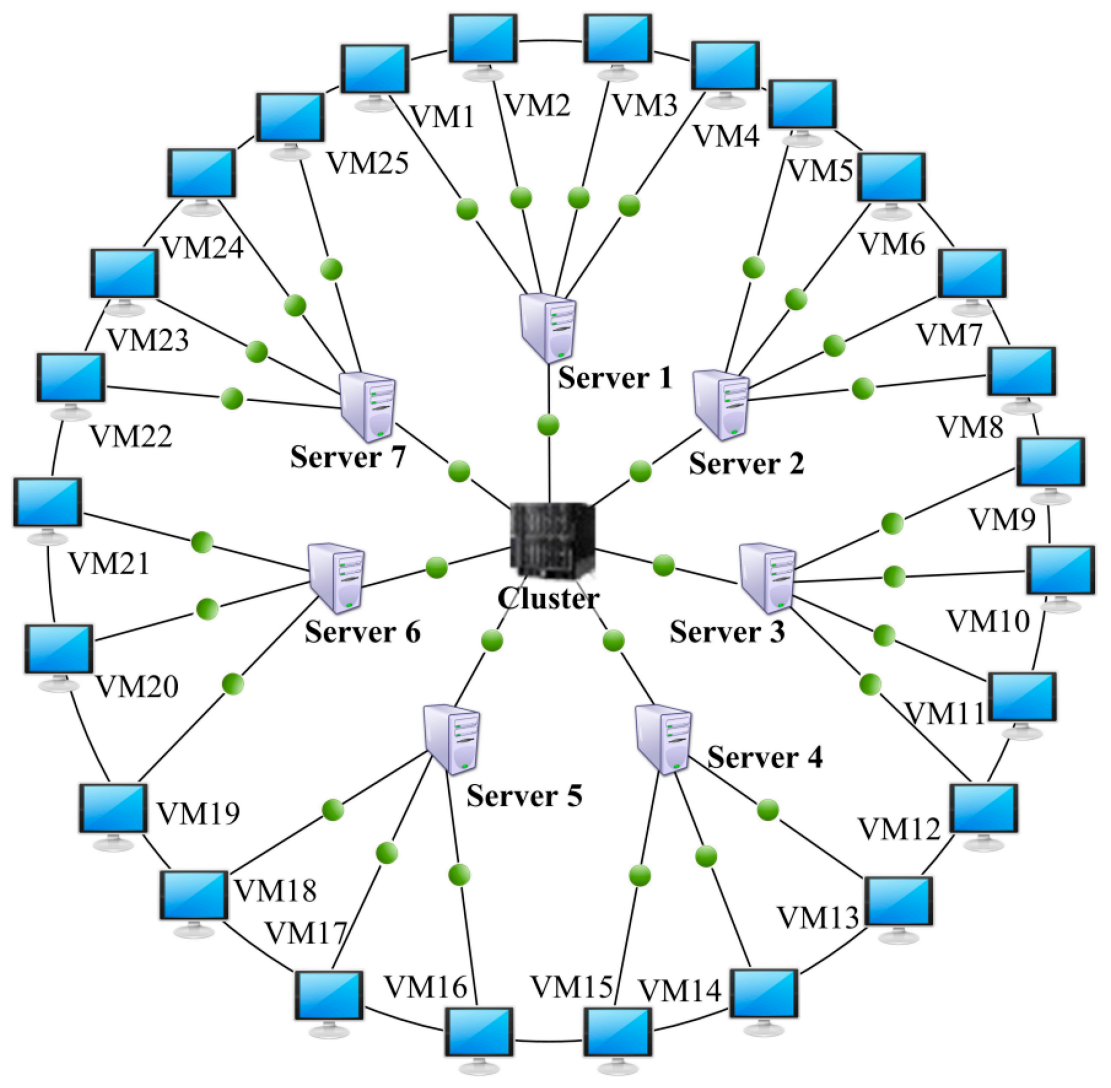
Cloud computing platform for the suggested GA program execution in parallel.

We have developed a parallel GA program based on MATLAB^®^ Distributed Computing Server (MDCS) and Parallel Computing Toolbox (PCT) to investigate the efficiency and performance of the suggested algorithm. In order to efficiently perform an in-depth experimental analysis, we use a cloud computing platform based on IBM PureFlex^®^ cluster with seven blade servers. In this cloud computing platform, 25 virtual machines have been built for searching optimal solution in parallel in our GA program, which is shown as Figure 5.

Based on the above experiment platform, we compared and analyzed the dependencies among the optimal reliability, the MAX service time, and the data security, as well as the robustness of the suggested algorithm, which are described in the following sections.

### 6.2. Optimal Reliability vs. MAX Service Time

In order to investigate the change of optimal reliability of a WSA along with the MAX service time rise under some given data security constraints, a set of experiments was designed. In suggested GA algorithm, the population size is set to 1600 chromosomes. The max generation is set to 30. The size of the optimal chromosome pool is dynamically increased from 0.5%–5% of population size with the increase of generation. The crossover probability is set to 0.7. The variation probability is set to 0.3. The penalty factor of reliability is set to 100. The number of repeated experiments is set to 20. A set of experiments were performed, which observe the searching process for the optimal reliabilities along with the MAX service time rise under the given data security constraints 0, 0.3, 0.6, 0.9, respectively.

#### 6.2.1. The Number of Experiments that Found Feasible Solutions

**Figure 6.**
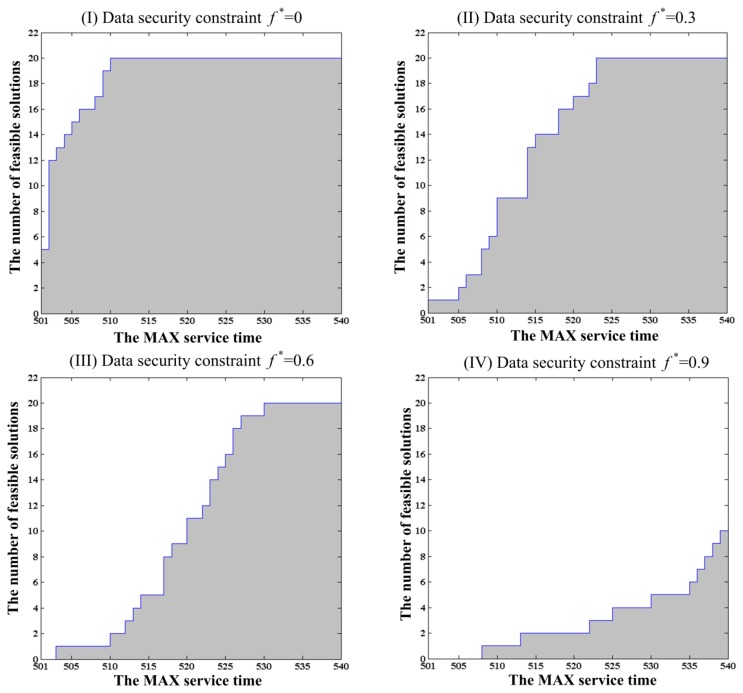
The change in the number of optimal solutions with alterations in the MAX service time from 501–540 under the different data security constraints.

For each MAX service time, the numbers of experiments that found feasible solutions in 20 repeated experiments is different under each constant data security constraint. The number of experiments that found feasible solutions for the MAX service time T* changes from 501–540 with a constant incremental change of 1 under the given data security constraints, which are shown in Figure 6.

From Figure 6, one can see that the number of experiments that found feasible solutions in 20 repeated experiments increases gradually along with the MAX service time. From the comparison of the curves of the number of feasible solutions in Figure 6I–IV, one can see that the climbing speed under a lower data security constraint is faster than that under a higher data security constraint. It indicates that it is more difficult to find plentiful feasible solutions under a stricter data security constraint. In addition, the shaded areas in Figure 6I–IV represent the number of feasible solutions subject to each given data security constraint. Comparing the area of shadows in Figure 6I–IV, one can draw an intuitive judgment that the amount of feasible solutions under a looser data security constraint is larger than one under a stricter data security constraint at the same MAX service time.

**Figure 7.**
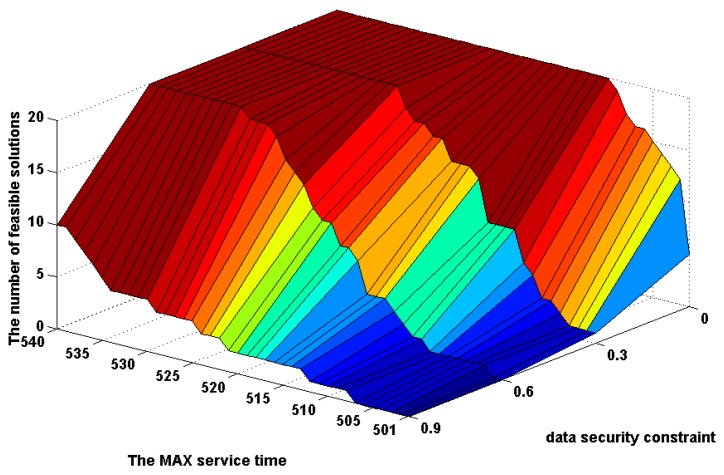
The change in the number of optimal solutions with alterations of the MAX service time from 501 to 540 and the data security constraint change from 0 to 0.9.

In order to investigate the relationship of the MAX service time and the data security constraint in the combining effects on the amount of feasible solutions, the above experimental results are shown in the form of 3D image in Figure 7. From this figure, one can see that the data security constraint plays the more important role in the increase of the amount of feasible solutions under a smaller MAX service time than one under a larger MAX service time. On the contrary, the MAX service time plays the more important role in the increase of the amount of feasible solutions under a stricter data security constraint than one under a looser data security constraint.

Thus, the conclusion can be drawn that the potential feasible solutions are found to be more difficult when the MAX service time is smaller or the data security constraint is stricter. Therefore, for a smaller MAX service time or a stricter data security constraint, it will take more genetic generations to search the potential feasible solutions.

#### 6.2.2. The Trend of the Optimal Reliabilities

**Figure 8.**
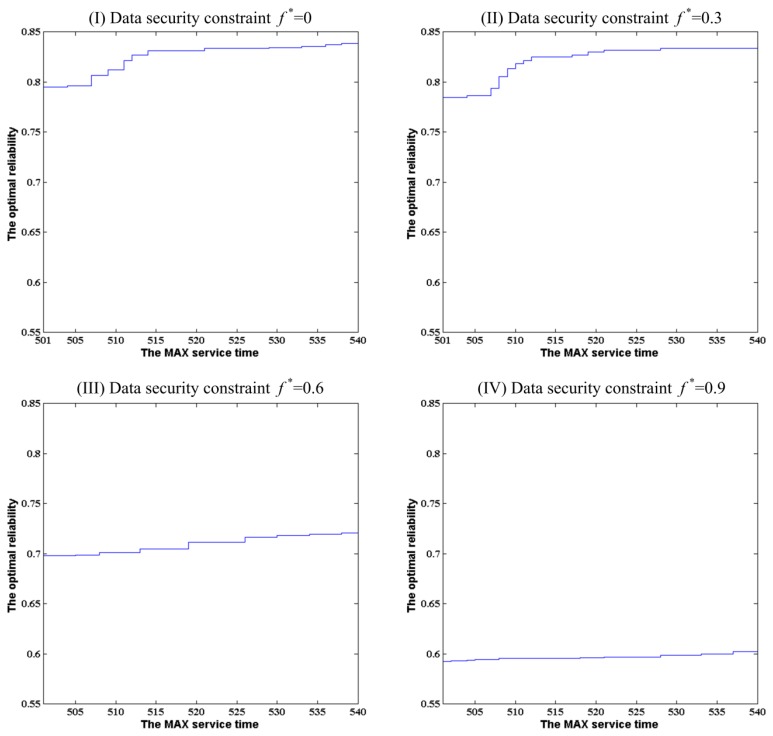
The change of optimal reliability along with the alterations of MAX service time from 501 to 540 under the different data security constraints.

Under each constant data security constraint, the change trend of the optimal reliabilities was investigated along with the MAX service time rise. The found optimal reliabilities for the MAX service time T* change from 501 to 540 with a constant incremental change of 1 under the given data security constraints are shown in Figure 8.

From Figure 8, one can see that the optimal reliabilities increase gradually along with the MAX service time. From the comparison of the curves of optimal reliabilities in Figure 8I–IV, one can see that the climbing speed under a lower data security constraint is faster than that under a higher data security constraint. It indicates that it is more difficult to find the solutions with higher optimal reliability under a stricter data security constraint. Similarly to Figure 6I–IV, the shaded areas in Figure 8I–IV represent the optimal reliabilities of solutions which are subject to each given data security constraint. Comparing the area of shadows in Figure 8I–IV, one can see clearly that the optimal reliability of feasible solutions under a looser data security constraint is larger than one under a stricter data security constraint at the same MAX service time. Additionally, it is more and more obvious along with the increase of data security constraint.

**Figure 9.**
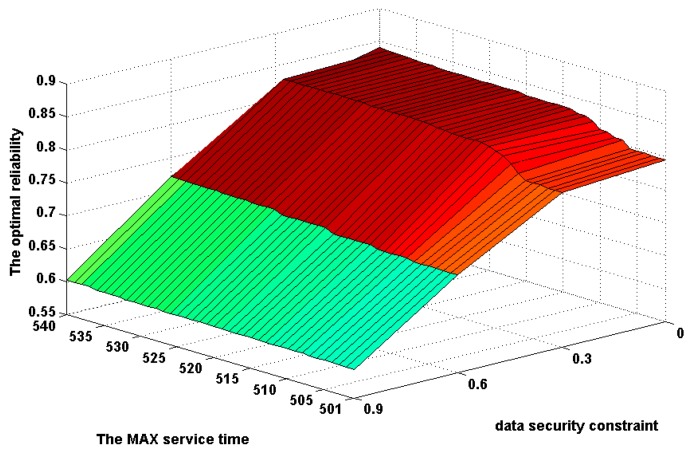
The change of optimal reliability along with the alterations of MAX service time from 501 to 540 and the data security constraint change from 0 to 0.9.

In order to investigate the relationship of the MAX service time and the data security constraint in the combining effects on the optimal reliability of feasible solutions, the above experimental results are shown as a 3D plot in Figure 9. From this figure, one can see that the data security constraint plays a more important role in the increase of the optimal reliability of feasible solutions than the MAX service time. On the contrary, comparing to the data security, the MAX service time plays a limited role on the increase of the optimal reliability of feasible solutions under each data security constraint.

Thus, the conclusion can be drawn that the potential feasible solutions are found to be more difficult when the MAX service time is smaller, or the data security constraint is stricter. Therefore, for a smaller MAX service time or a stricter data security constraint, it will take more genetic generations to search the potential feasible solutions.

The reasons for the above results are that the probabilistic sum of optimal reliabilities of the feasible solutions changes more violently along with the alterations of the data security constraint than with the alterations of the MAX service time. It indicates that the reliability of WSA is largely dependent on the restriction of data security constraint. Therefore, determining an appropriate data security constraint is very important to improve the reliability of WSA. When the data security constraint is determined, the reliability of a WSA can be enhanced to a certain extent by choosing an appropriate MAX service time.

#### 6.2.3. The Trend of the Mean Value of Optimal Reliabilities

**Figure 10.**
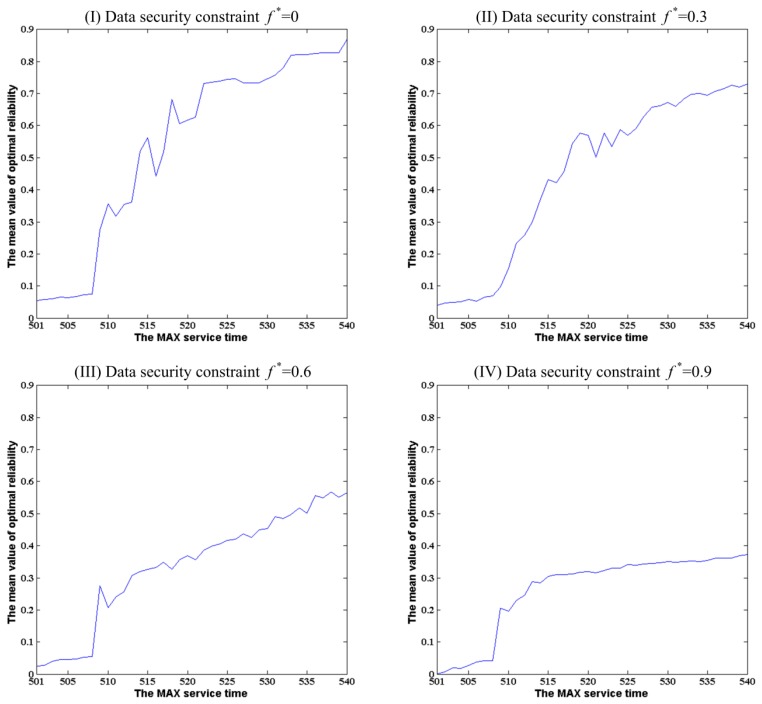
The mean values of optimal reliability along with the MAX service time change from 501 to 540 under the different data security constraints.

**Figure 11.**
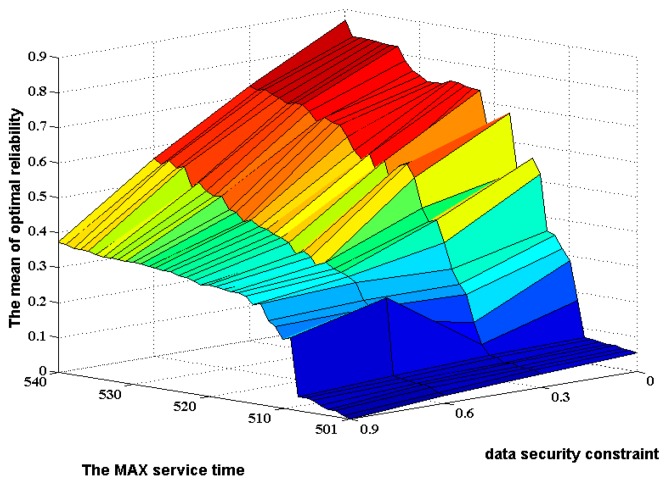
The mean values of optimal reliability along with the MAX service time change from 501–540 and the data security constraint change from 0 to 0.9.

In addition, we also investigated the mean values of the optimal reliabilities in 20 repeated experiments. Figure 10 shows these mean values for the MAX service time T* change from 501 to 540 with a constant incremental change of 1 under each constant data security constraint. The curves of the mean value of optimal reliability in Figure 10 from I–IV show a growth trend on the whole along with the MAX service time rise. The 3D plot of the mean value of optimal reliability is shown in Figure 11.

In such a situation that the exact values of reliability need not be calculated, the mean values of the optimal reliabilities in an adequate number of repeated experiments still have a role in comparing the different service distribution solutions. Obviously, the maximum number of generic generations in these repeated experiments can be far less than those in the case of calculating exact reliabilities. Therefore, these mean values can be used for the high effective determination of the appropriate MAX service time and the appropriate data security constraint in the rough range according to the climbing speed of curves in Figures 10 and 11.

Thus, the reliability, the data security and the MAX service time are contradicting optimization objectives. The suggested algorithm allows one to find the compromise solution by changing the constraints and solving the optimization problems (1) or (2). Another possible approach is solving the multi-objective optimization problem with reliability, security and MAX service time as different criteria. Based on the above analysis, we suggest a distinct solution method for problem (1) or (2) in the next section.

### 6.3. A Distinct Solution Method for the Problems (1) or (2) in Section 3.3

**Figure 12.**
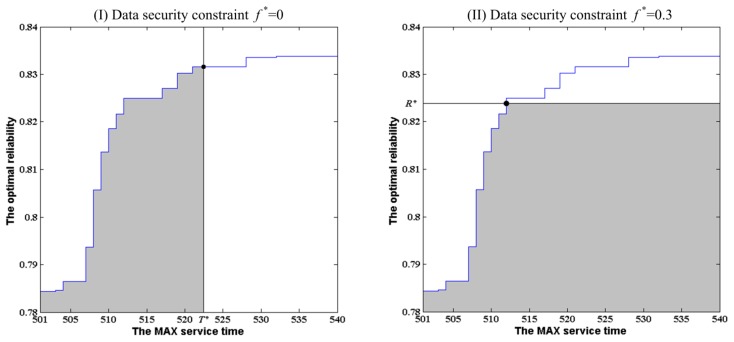
The distinct solution method for the problem (1) and (2).

On the basis of the above experimental analysis, we present a distinct solution method for the problems (1) or (2) described in Section 3.3 based on Figure 8 in Section 6.2.2. For the problem (1), based on Figure 8 (II) corresponding to the given data security constraint assuming $f_{\text{φ}}^{*}=0.3$, we can draw a vertical auxiliary line according to the given MAX service time value T*, which is shown in Figure 12I. The intersection between the vertical line and the optimal reliability curve forms a shadow area. The points fallen into the shadow area represent the feasible solutions subject to *T*≤T* and $f_{\text{φ}}\leq f_{\text{φ}}^{*}$. The point with maximal reliability in these points is just the optimal solution. Obviously, the point is the cross point of the vertical line and the optimal reliability curve. Thus, the reliability corresponding to the point is just the solution of the problem (1).

For the problem (2) described in Section 3.3, the distinct solution method is similar to the above description. According to the given R*, we can draw a horizontal auxiliary line corresponding to the given reliability value R* based on Figure 8II, which is shown in Figure 12II. The intersection between the horizontal line and the optimal reliability curve forms a shadow region. The points fallen into the shadow region represent the feasible solutions subject to *R*≥R* and $f_{\text{φ}}\leq f_{\text{φ}}^{*}$. The point with the minimal value of MAX service time in these points is just the optimal solution. Obviously, the point is the cross point of the horizontal line and the optimal reliability curve. Thus, the MAX service time corresponding to the point is just the solution of the problem (2).

In the case that the restrictions of the MAX service time and data security constraint are too strict, the auxiliary line cannot cross over the optimal reliability curve. Thus, a feasible solution of problem (1) or (2) cannot be found. In such a case, the improvement of reliability or the decrease of expected service time of WSA can be obtained only by increasing the amount of SNs available for the execution of ASs in parallel. The influence of the number of SNs on the optimal reliability of WSA is further invested in the next section.

### 6.4. Optimal Reliability vs. the Number of SNs

In order to investigate the influence of the number of SNs on the optimal reliability, a set of experiments were designed. The variation range of the number of SNs is set from 6 to 15 with a constant incremental change 1. In order to compare the influence of different number of SNs on the reliability of WSA, the SNs gradually added to the WSN service system available for the WSA are set to the same. The MAX service time and the data security constraint are set as 620 and 0.4, respectively. Other parameters of our GA algorithm are set as the same as Section 6.2.

**Figure 13.**
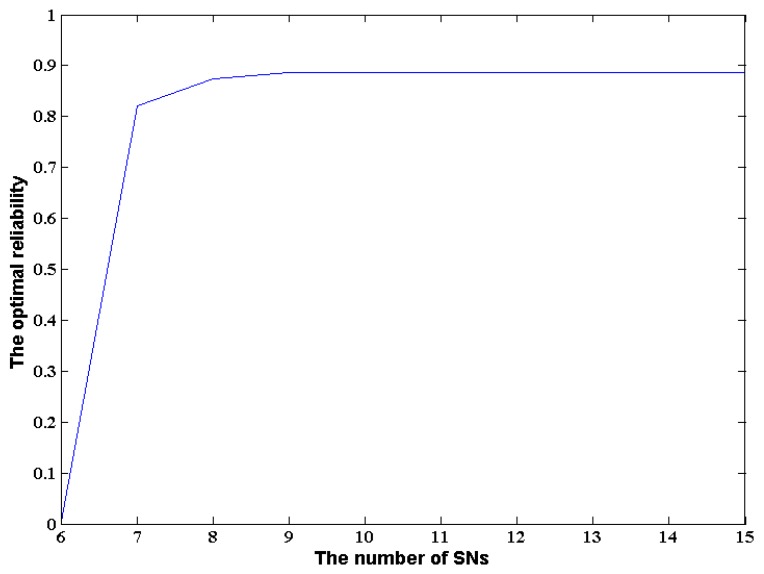
The change of optimal reliability along with the increase in the amount of SNs.

The experiments were performed 30 times. Figure 13 shows the change of optimal reliability of WSA along with increasing the number of SNs from 6 to 15.

From Figure 13, one can see that the reliability closes to zero when the number of available SNs is 6. After adding one SN to the WSN service system, the reliability rapidly increases to 0.8219. Along with the increase of the number of SNs, the reliability slowly but steadily climbs to the pinnacle: 0.8859. After that, the reliability remains unchanged even when SNs are continued to be added to the WSN service system. This result indicates that increasing the number of SNs can only enhance the reliability of WSA to a certain degree when the MAX service time and data security constraints are given values. Obviously, the optimal configuration amount of SNs can be found directly from Figure 13. Therefore, the intersection of the auxiliary line and the optimal reliability curve can be created by improving the reliability or by decreasing the expected service time though increasing the appropriate number of SNs. On this basis, problems (1) and (2) can be solved by employing the distinct solution method proposed in the previous section. In addition, the suggested method is also helpful to reduce waste of computing resources and improve their utilization efficiency as much as possible.

## 7. Conclusions

The WSA is a newly developed computing model of delivering WSN services in the Internet of Things. The WSA model allows effective distribution of computational tasks among different SNs available in the WSN service system [40,41]. The WSB can map the user's service request into a set of ASs and send them to some different SNs for parallel execution [42,43]. In order to provide a desired level of service reliability, the WSB can assign the same ASs to several different SNs. However, executing the same ASs at different SNs as well as sending multiple replicas of associated data through different channels increases the probability of unauthorized access to sensitive data, which leads to reduction of the service data security. For any service request from a user, the service reliability, service completion time and data security depend on service task partition into ASs and their distribution among the available SNs. The suggested optimization algorithm is aimed at achieving the maximum reliability or minimum service completion time subject to data security constraints by the optimal service task partition and distribution. The high computational efficiency of the proposed algorithm is achieved using the universal generating function approach. This allows the algorithm to be used in optimization procedures where a large number of different solutions should be estimated.

## Figures and Tables

**Table 1.** Parameters of SNs and their communication channels.

# of SNs (channels) *l*	*x_l_* (megabytes s^−1^)	*λ_l_* (s^−1^)	Security *y_l_*	*s_l_* (mega operations s^−1^)	*π_l_* (s^−1^)
1	10	0.0001	0.99	20	0.0001
2	4	0.0003	0.85	14	0.0002
3	6	0.0004	0.82	13	0.0010
4	2	0.0002	0.88	12	0.0003
5	1	0.0002	0.83	10	0.0002
6	5	0.0002	0.85	15	0.0001
7	4	0.0003	0.92	12	0.0001
8	9	0.0001	0.94	18	0.0002
9	5	0.0002	0.94	11	0.0003
10	2	0.0002	0.90	11	0.0002

**Table 2.** Service partition and parameters of ASs.

# of Sub-Services *i*	# of ASs *j*	*c_j_* (Mega Operations)	*I_j_* (Megabytes)	*O_j_* (Megabytes)	With Security Requirement
1	1	100	5	12	FALSE
1	2	1500	12	7	FALSE
2	3	200	7	3	TRUE
2	4	500	3	8	TRUE
3	5	1200	8	10	TRUE
3	6	300	10	7	TRUE
